# Surgical site, gender, and place of residence influence the time to resume driving after total joint arthroplasty

**DOI:** 10.1186/s40945-021-00111-4

**Published:** 2021-06-29

**Authors:** Tiberiu-Adrian Lazar, Martin Edelmann, Friedemann Awiszus, Christoph H. Lohmann

**Affiliations:** 1 Department of Orthopedic Rehabilitation, Celenus Fachklinik Teufelsbad, 18 Michaelstein Strasse, 38889 Blankenburg, Germany; 2 Department of Orthopedic Surgery, Otto-von-Guericke University, 44 Leipziger Strasse, 39120 Magdeburg, Germany

**Keywords:** Total knee arthroplasty, Total hip arthroplasty, Motor vehicle driving, Rehabilitation

## Abstract

**Background:**

For a large proportion of the population, especially those residing in the countryside, the use of a car for daily activities is indispensable. Following a TKA or THA procedure, the overseeing physician will usually recommend refraining from driving, sometimes up to 12 weeks after surgery with a major social and economical impact on patient’s life.

**Objective:**

Considering the legal stipulations in Germany regarding fitness to drive a motor vehicle, the aim of this study is to determine the time point when patients after total knee arthroplasty (TKA) or total hip arthroplasty (THA) take up driving again postoperatively. Further, we assessed the replaced joint, side, gender, place of residence and physician’s recommendations influencing the patient in making the decision to start driving again.

**Methods:**

92 eligible participants, contained within the frame of a prospective experimental observational study, were contacted via telephone 12 weeks after surgery and interviewed using a structured questionnaire. The answers were statistically analysed using SPSS® Version 26 for Windows.

**Results:**

Male participants resumed driving between the 6th and 7th week post-surgery, female participants resumed driving between the 8th and 9th week post-surgery. For 58.6% of patients the reason for the first post-operative use of a vehicle was medical: the journey to physical therapy or to a doctor’s appointment. There were statistically significant differences regarding operated side, gender and place of residence. TKA impaired patients the most. Patients recovering from a TKA drove considerably later. Patients recovering from a right sided TKA had an increased risk (9 times) not to become an “early driver”. Female patients who underwent TKA had an increased risk by a factor of 21 of becoming a “late driver”. In the ageing population, surgeons, physical therapists and rehabilitation professionals need to consider new approaches in providing options for patients’ mobility. Interestingly, there is a different need for early use of own vehicle in rural regions whereas in cities patients start driving later. There are clear differences between gender and surgical site.

**Conclusions:**

The rehabilitation following a right sided TKA proved a challenge with regard to the reuptake of driving. This should be taken into account when planning the course of therapy for patients who are driving regulary. Female patients could benefit from special training.

**Trial registration:**

retrospectively registered, DRKS00018693 https://www.drks.de/drks_web/navigate.do?navigationId=trial. HTML&TRIAL_ID=DRKS00018693.

**Supplementary Information:**

The online version contains supplementary material available at 10.1186/s40945-021-00111-4.

## Background

Osteoarthritis of the knee and hip joints is a common disease worldwide. In the GEDA 2014/2015-EHIS study conducted by the Robert-Koch-Institute of Germany, 17.9% of adults above the age of 18 report having suffered from arthritis in the last 12 months [1].

Replacement of the knee or hip joint following progressive joint destruction and function loss due to osteoarthritis has a major social and economical impact on patients’ life, being accountable for production downtime costs of 18.5 billion Euros and over 10 million days of sick leave in 2018 in Germany [2, 3].

According to the Endoprosthesis Register in Germany (ERPD), approximately 450.000 endoprosthetic procedures (primary and revision arthroplasty) were performed in 2018 [4]. Further, a search was performed using the database of the German federal office of statistics (DESTATIS) using the following string: “Koxarthrose/Gonarthrose” as a discharge diagnosis from hospital in 2018. This identified 178.329 patients with osteoarthritis of the hip and 185.439 patients with osteoarthritis of the knee [5].

Driving a car is important for patients in general because it provides the opportunity for personal control and autonomy. This is especially important for those residing in the countryside due to a lack of public transport. For individual with difficulties in walking or riding a bike due to health issues, driving a car is often the only option for an independent mobility, it is often necessary to make doctors’ and physical therapists’ appointments and sometimes it is a requirement to resume working not just in the remote countryside [6].

During the postoperative rehabilitation phase, one of the most frequently asked questions, by patients following a TKA or THA is “When can I start driving again?” In Germany, health care insurance providers are legally mandated to finance a three-week in-patient postoperative rehabilitation (bedridden or outpatient) following TKA or THA. Because most of the patients with a TKA or THA undergo a bedridden rehabilitation and they will be discharged between the 4th and 5th week after surgery, there is no reason to drive a motor vehicle before their discharge. Also, the legal stipulations in Germany require that every driver must evaluate whether they view themselves well enough to drive prior to starting their engine. The driver must evaluate to what extent he/she can fulfil the requirement for roadworthiness. Doctors’ recommendations should guide and support the patients in this evaluation. The decision to get behind the wheel of a motor vehicle, however, is made by the patient – that is what the legislation stipulates.

During driving it is of utmost importance to have the ability to brake in time to avoid an obstacle. Both the cognitive and the motor skills are important. The interaction of these skills constitutes the brake reaction time, which represents a significant parameter of safe driving. That is why a large number of studies [9–13] make use of this parameter. A brake reaction time within the normal range offers a decision-making tool to determine the time to start driving again. The measured brake reaction time is a parameter of safe driving, however not the main criteria for postoperative roadworthiness.

Regarding recommendations when to start driving after TKA or THA, there are a number of studies published [7–19]. The literature shows that there is a very large bandwidth of recommendations when to start driving after TKA or THA, ranging from two days up to 8 weeks after surgery [7, 8, 12–19]. In Germany in some cases the recommendation to restrain from driving after TKA or THA may be up to 12 weeks after surgery.

The aim of the current study was to determine when patients after TKA or THA actually start driving again after surgery. Further, we assessed if the replaced joint, the side, gender, place of residence and potential physician’s recommendations influence the patient in the decision making process when to start driving again.

## Methods

The present prospective experimental observational study was conducted as a single centre study in an orthopaedic rehabilitation clinic in Germany following the guidelines “The Strengthening the Reporting of Observational Studies in Epidemiology” (STROBE) statement.

For the statistical analysis the significance level was set for 5%, while the confidence interval was set for 95%. The ideal sample size was calculated to be 90 participants [20].

The study was approved by the ethical committee of Sachsen-Anhalt’s medical board (No. 18/19). The application to register the study with the German clinical trials register (DRKS) was sent on the 30th of august 2019 and the registration process was completed on the 20th of September 2019.

100 participants were included in the study and provided informed consent prior to inclusion in the study, all of whom had been referred to the Celenus Fachklinik “Teufelsbad” Blankenburg for in-patient rehabilitation from five surrounding hospitals. Recruitment occurred between July 2019 and November 2019. In this time frame the authors oversaw the rehabilitation of 167 patients following TKA or THA. Of these, 59% were recruited into the study. This sample size fulfils criterion number 3 of the Quality Assessment Tool for Observational Cohort and Cross-Sectional Studies [21].

Patients were included following the criteria: primary TKA or primary THA, age between 20 and 90 years, a valid driver’s license, and regular driving practice. Exclusion criterion: total hip joint replacement surgery following trauma. Because all study participants had regular driving practice prior to surgery, comorbidities were not taken into account for this study.

The questionnaire to collect the data was created specifically for this study and was based on the aim of this study. It included data about participants driving licence, age, gender, place of residence, operated joint and side, date of the operation, use of walking aids, driving practice, time frame of postoperative driving resumption (weeks), pain level, car’s transmission type and the reason for a first driving activity after surgery.

The authors decided to determine a 12 weeks period as the maximum amount of recovery time after a TKA or THA procedure with no further complications. Beginning with the 13th post-operative week participants were contacted via telephone, and the study questionnaire was filled out by the investigator according to the answers given by the participant.

**Fig. 1:**
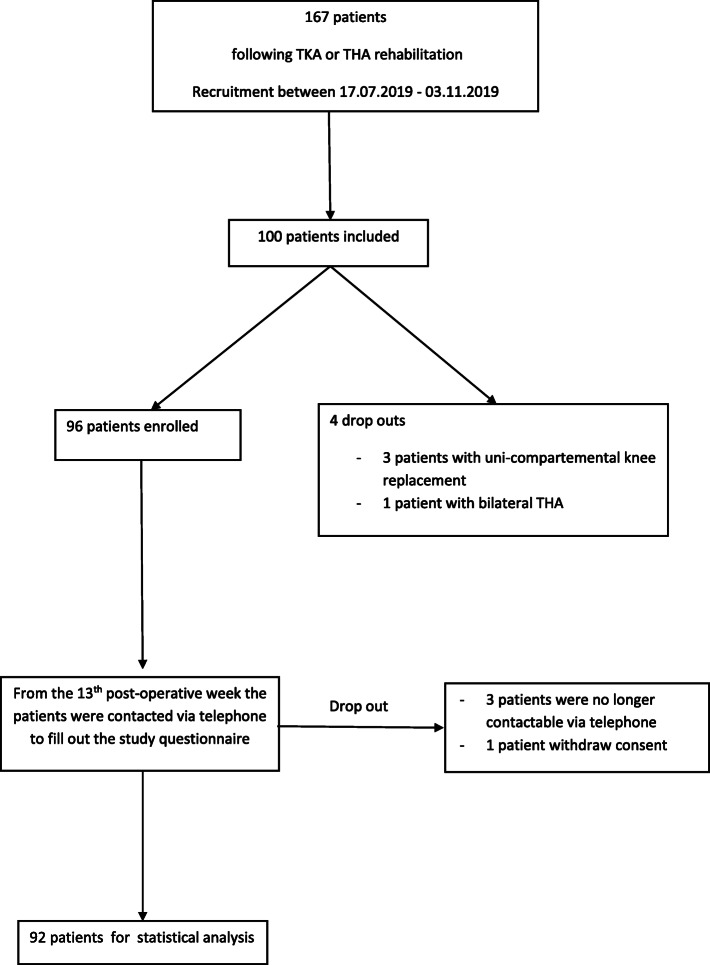
Flowchart patient enrolment

One participant revoked his consent, three other participants could not be contacted via telephone. Four participants showed false recruitment (three patients with uni-compartmental knee replacement and one patient with bilateral total hip arthroplasties) and these individual were excluded from the study. Finally, one participant had to be excluded since the place of residence was missing. Of the 100 original participants 92 remained who fulfilled the criteria and were included in the study. The drop-out rate was 4,1%. (Flowchart patient enrolment, Fig. 1).

The study sample (Table 1) consisted of 56 male and 36 female participants with an age range of between 34 and 81 years of age. The Mean age for male participants was 64.2 years of age, for female participants it was 60.6 years of age.

**Table 1:** Study sample

	Male	Female	Total
right TKA	11	6	**17**
left TKA	14	11	**25**
right THA	16	10	**26**
left THA	15	9	**24**
Total	**56**	**36**	**92**

### Statistical analysis

Descriptive statistics and all the analyses were completed using SPSS® Statistics Version 26 software for Windows from IBM Corporation and Excel® from Microsoft Corporation. We assumed that the collected data had an equality of variances. According to this assumption we used an ANOVA analysis. Gender, operated side, location of residence, vehicle transmission type and the reason for the first postoperatively drive, regarding the reuptake of driving postoperatively were analysed using Levene’s test, t-test, Pearson chi-squared test and Fisher’s exact test. A ***p*** value < 0.05 was considered statistically significant. 95% confidence intervals (CIs) were estimated for each variable and odds ratio analysis was conducted for the interaction between operated side/joint and gender/joint.

## Results

92.4% (*n* = 85) of the participants drove within the first 12 weeks of recovery following surgery. Seven participants refrained from driving – one participant suffered from an apoplexy in the first 12 weeks postoperatively. Six participants answered the question “Did you resume driving after your operation?” with “I have not felt fit enough to drive (yet)”. None of the participants who drove reported any accidents in the first 12 weeks of recovery.

29.3% (*n* = 27) of the participants resumed driving within the first 6 weeks after surgery. The earliest resumption of driving was within the 3rd postoperative week

For 58.6% of the participants (*n* = 54) the first reason for driving was health-related: driving to physical therapy or a doctor’s appointment.

**Fig. 2:**
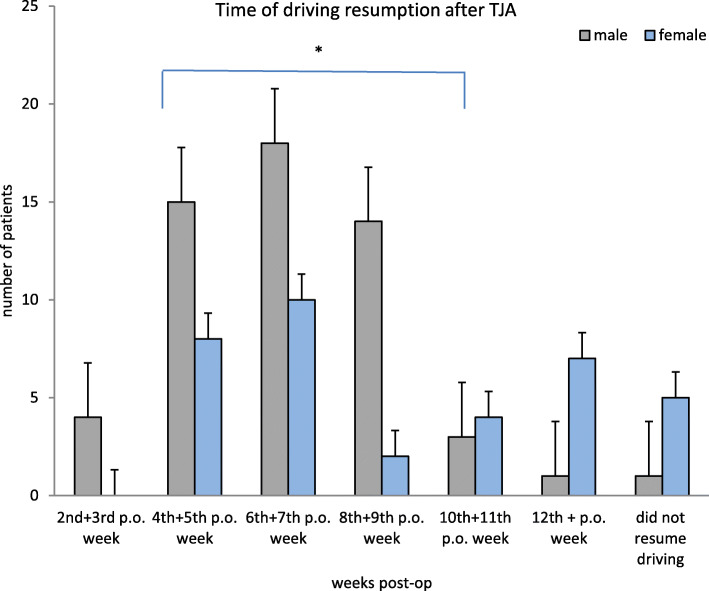
This figure displays the time of driving resumption after TJA. The majority of the patients started to drive between the 4th–9th postoperative weeks

The gender analysis of the postoperative resumption of driving showed that male participants resumed driving earlier than female participants (*p* = 0.0015). (Fig. 2).

**Fig. 3:**
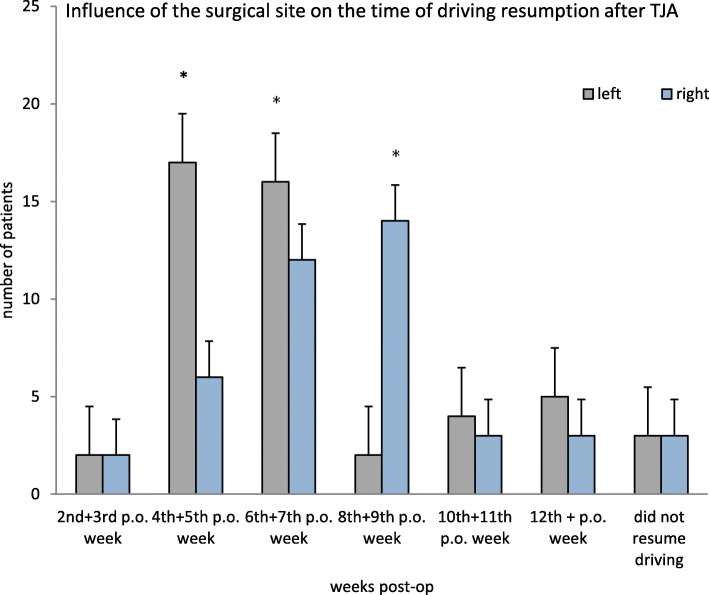
This figure displays the influence of the surgical site on the time of driving resumption after TJA. The majority of the patients started to drive between the 4th–9th postoperative weeks. Significant more patients started to drive during 4th – 7th postoperative weeks when the left side was operated on

The analysis of the operated side (left vs. right) showed a significant side preference (*p* = 0.01) in the postoperative resumption of driving. Participants who underwent a TKA or THA on the left side resumed driving earlier than participants who underwent a TKA or THA on the right side. (Fig. 3).

The Mode for the left side was in the 4th + 5th postoperative week, while the Mode for the right side was in the 8th + 9th postoperative week, with 17 and 14 participants, respectively.

**Fig. 4:**
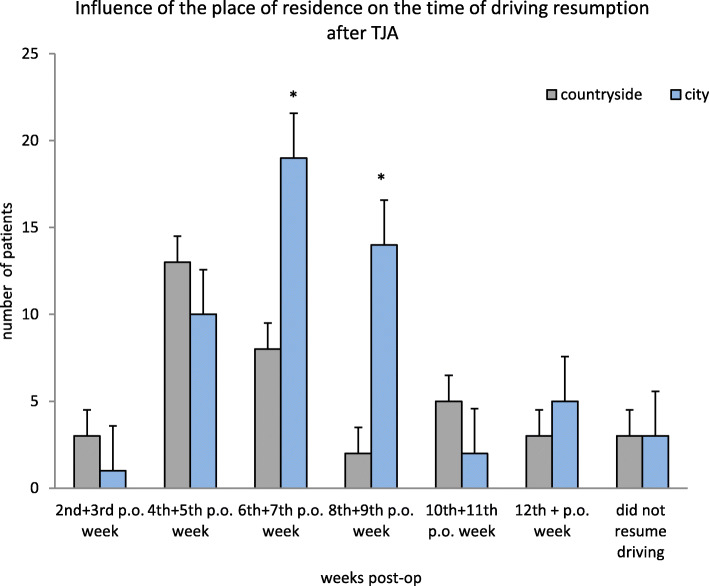
This figure displays the influence of place of residence on the time of driving resumption after TJA. The majority of the patients started to drive between the 4th–9th postoperative weeks. Significant more patients residing in the cities started to drive during 6th – 9th postoperative weeks compared to patients residing in the countryside who started early to drive again

The location of residence also had a statistically significant effect on the time frame where the participant resumed driving (*p* = 0.02). Participants who lived in the countryside drove earlier than participants who lived in cities. (Fig. 4).

The Mode for participants who resided in the countryside is in the 4th + 5th week post-surgery, while the Mode for participants who resided in cities is in the 6th + 7th week post-surgery, with 13 and 19 participants, respectively.

In our statistical analyses, we were unable to find a significant difference (*p* = 0.05) between TKA and THA regarding the time frame when the participants resumed driving.

**Fig. 5:**
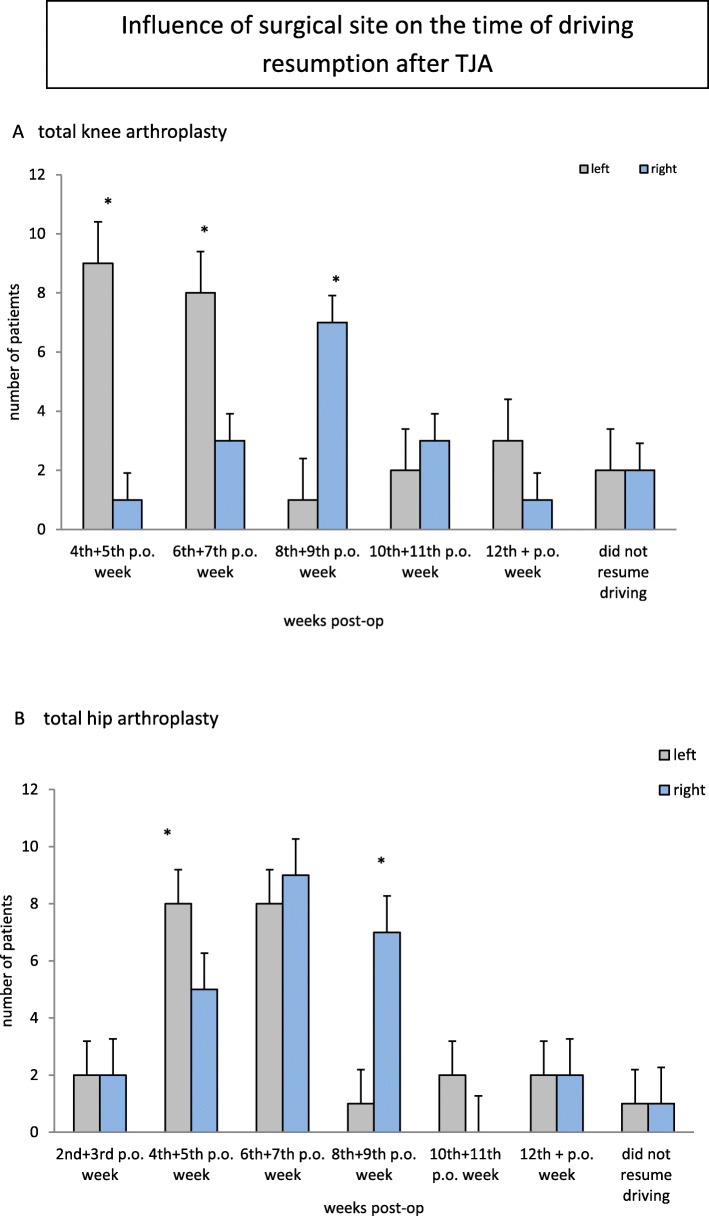
This figure displays the influence the surgical site (left or right) for TKA (**A**) or THA (**B**) on the time of driving resumption after TJA. The majority of the patients started to drive between the 4th–9th postoperative weeks. Significant more started to drive during 4th – 6th postoperative weeks when receiving a TKA on the left side (**A**). This was similar for THA (**B**), although patients receiving a THA on the left side already started to drive earlier (6th + 7th postoperative weeks)

The interaction between the operated side and operated joint was also analysed which showed a significance for TKA (*p* = 0.02) but no significance for THA (*p* = 0.3). Participants with left-sided TKA resumed driving earlier than participants with right-sided TKA. (Fig. 5).

Additionally, we conducted two risk analyses: The risk analysis for side and joint presented statistical significance for TKA (*p* = 0.03). Regarding the Odds Ratio analysis for operated side, participants who were operated on the right side were 9.0 times less likely to be a “early driver” (resumption of driving before the 6th + 7th week post-surgery; 95% CI 1.01–79.54). The risk analysis for gender and joint showed also statistical significance (*p* = 0.00015); female participants who had undergone a TKA were 21.08 times more likely to be “late drivers” (resumption of driving 10th + 11th weeks post-surgery; 95% CI 3.64–121.83). Therefore, female patients and patients who underwent a right-sided TKA can be considered high risk groups to be a “late driver”.

A correlation between place of residence and reason for the first drive post-surgery could not be statistically supported (*p* = 0.92).

Moreover, there was no statistical significance (*p* = 0.26) showing that vehicle transmission type (manual or automatic) affected the timeframe when participants resumed driving after TKA or THA procedures.

Most of the study participants still experienced some discomfort but no leg pain during daily activities 12 weeks after joint replacement. However, 92.4% (*n* = 85) of the participants who had resumed driving after TKA or THA reported that they had no joint or leg pain when pushing down the car pedals (data not shown).

## Discussion

A main goal of a patient undergoing TKA or THA procedure who had pre-operative regular driving practice, is the swift resumption of driving after surgery. Patients who wish to drive again early after surgery should receive individualised recommendations and precise support.. In our study, we investigated when patients start driving after TKA or THA. We further assessed if the replaced joint, the side, gender, place of residence and potential physician’s recommendations influence the patient in the decision making process when to start driving again.

Male participants resumed driving between the 6th and 7th week post-surgery, female participants resumed driving between the 8th and 9th week post-surgery. This study aids to substantiate that different study sub-groups partly show clearly different results. For this reason, recommendations should be individualised because “one size fits all” recommendations are not helpful in this context.

A striking result of the gender analysis of postoperative driving was the binomial distribution of female participants. Male participants and a proportion of female participants behave similarly. A smaller proportion of female participants resumes driving later, in the 10th–12th week post-surgery. Thus, a specific training and individualized support for patients who may be “late drivers” such as females and a right TKA should be considered.

Another result of the study was the discrepancy between study participants who reside in the countryside and participants who reside in the cities with respect to the timing of first driving activity following surgery. The authors defined “city” with a population of ≥30.000 and “countryside” with a population of < 30.000. The number of inhabitants resemble a mid-size German town, however, it showed statistically significant differences with respect to the time point when patients resumed driving post-surgery. We assume that “cities” provide a better infrastructure for public transportation, which can be used and thus reduces the need for a car. Participants who reside in the “countryside” resumed driving considerably earlier (the mode in the “countryside” was 13 participants in the 4th + 5th week post-surgery) than participants who reside in the “cities” (the mode was 19 participants in the 6th + 7th week post-surgery). 58.6% of the participants gave a health care reason for the first post-operative drive: such as travel to physical therapy or a doctor’s appointment. A hypothesised correlation between size of residential location and reason for the first post-operative drive could not be statistically confirmed (*p* = 0.927).

In a number of studies [7, 9, 12–15] the operated side did not influence the postoperative begin of driving activities. Our results show a different picture: there was no trend in patients who had undergone THA (*p* = 0.304), however patients who had undergone right-side TKA drove significantly later (*p* = 0.020). A possible explanation for this is that driving requires only limited hip movement, whereas the right knee has the key function for acceleration and pushing the brake pedals. Thus, roadworthiness largely depends on the functionality and performance of the right knee. Clearly, this aspect is very central for the decision of roadworthiness the patient must make prior to driving.

In a study by Rondon et al. 98.2% of the study participants resumed driving within the first 12 weeks post-surgery [8]. We were able to deliver similar results, 92.4% of our study participants resumed driving in the first 12 weeks post-surgery.

In our study, the average time frame in which participants resumed driving following a TKA or THA was between the 6th and 7th week post-surgery. We were not able to find a significant difference between TKA and THA (*p* = 0.053). In contrast, Rondon et al. discovered a statistically significant difference between knee and hip arthroplasties (TKA = 4.4 weeks, THA = 3.7 weeks) [8].

This discrepancy could be explained by two possible reasons. In Germany, health insurance providers are legally mandated to finance a three-week in-patient postoperative rehabilitation program following total knee or hip arthroplasty. Our participants were recruited from the patient population in the rehabilitation facility and were therefore all undergoing a three-week in-patient rehabilitation, they were discharged between the 4th and 5th week post-surgery. For our study participants there was therefore no reason to drive before their discharge date. In addition to this, the sample size in this study was considerably smaller (*n* = 92) than the sample size in the study conducted by Rondon et al. (*n* = 1044) [8]. This may explain why we were unable to find a statistically significant difference between total knee and hip arthroplasties with regard to the resumption of driving post-surgery. Rondon et al. identified the implementation of postoperative rehabilitation as a reason for later resumption of driving [8].

We identified a higher risk of being a “late driver” if the participant received a right-side procedure or if the participant was female. Rondon et al. delivered similar results; patients with surgery of the right side and female patients drove later [8]. In addition, we conducted an Odds Ratio test regarding to the risk of resuming driving later post-surgery. An “early driver” was defined as a participant who resumed driving in the 4th + 5th week post-surgery. A “late driver” was defined as a participant who resumed driving in the 10th + 11th week post-surgery. There was an increased risk for patients who had undergone a right-sided TKA not to resume driving in the 4th + 5th week post-surgery. The risk analysis for gender and joint revealed a 21.08-fold increased risk (95% CI 3.64–121.83) of female patients who had undergone TKA resuming driving in the11^th^ + 12th week post-surgery. It therefore appears that female participants may need the recommendation given by doctors more than male participants. Consequently, this implies that female patients who have undergone a TKA should receive specific guidance and a more focussed rehabilitation program if they intend to drive post-surgery. 13.9% (*n* = 5) of the female participants did not drive in the 13th week pot-surgery compared to 1.8% (*n* = 1) of the male participants.

Davis et al. (2018) showed that both male and female participants (*n* = 32) achieve their preoperative brake reaction time after the 2nd postoperative week [9]. Most of the participants drove with an automatic transmission. The operated side did not have an impact on how fast the preoperative brake reaction time was achieved whereas gender did: male participants reached their preoperative brake reaction earlier than female participants [9].

In Germany, a driver must evaluate his own roadworthiness before he can start his engine. Roadworthiness is defined as the ability to safely operate the vehicle, regardless of situation. Limitations of roadworthiness, example e.g. drug or alcohol consumption are prosecuted by the legislator. However there are no legislative specifications regarding temporary physical limitations. The driver must evaluate himself and refrain from driving if he does not feel roadworthy. This self-evaluation is therefore crucial from a legal standpoint. The present prospective study examines this self-evaluation with regard to roadworthiness.

The mean age of our male participants was 64.2 years, while the mean age of our female participants was 60.6 years. The 65–74-year-old participants make up the group of drivers with the lowest accident rate at 2.86 accidents per 1000 drivers per year [22]. If extrapolated to the 3-month span, this would result in 0.715 accidents per 1000 drivers. Rondon et al. reported 0.9% accidents in the 3-month period for knee arthroplasties and 0.4% accidents in the 3-month period for hip arthroplasties [8]. A statistical comparison was not feasible since the accident rate was not elevated in drivers who had undergone TKA or THA when compared to the general age population. Our participants did not report any accidents. From this we may assume that the self-evaluation of roadworthiness following TKA and THA was adequate and the individual time frame chosen by the participants to resume driving, was responsibly chosen.

After TKA or THA patients experience considerable limitations of the function of their affected leg for an uncertain period of time. This is why they turn to their overseeing doctor or physical therapist, for recommendations regarding post-op resumption of driving. A glance at the current research literature as an anchor or orientation regarding this recommendations, does not deliver consistent evidence.

The available studies can be categorised into two groups: studies which record when patients drive post-surgery and respect their participants’ self-evaluations and studies which use measurable parameters, most often brake reaction time, to recommend when patients can resume driving post-surgery (Tables 2 and 3).

**Table 2:** Recommendations to reuptake driving after TKA or THA based on patients self evaluation

Study	THA	TKA
Quarashi et al. [12]	2 days p.o.	–
Batra et al. [7]	1 week p.o.	–
Van der Velden et al. [13]	2 weeks p.o.	4 weeks p.o.
Rondon et al. [8]	3.7 weeks p.o.	4.4 weeks p.o.
Elanti et al. [14]	–	6 weeks p.o.
Latz et al. [15]	2–4 week p.o.	2–4 weeks p.o.
Goodwin et al. [16]	6–8 week p.o.	10 days – 8 weeks p.o.
This study	6th + 7th week p.o.	6th + 7th week p.o.

**Table 3:** Recommendations to reuptake driving after TKA based on break reaction time

Study	Right TKA	Left TKA
Marques et al. [17]	44+/− 19 days p.o.	20+/− 15 days p.o.
Nizam et al. [18]	3 weeks p.o.	3 weeks p.o.
Van der Velden et al. [13]	4 weeks p.o.	4 weeks p.o.
Huang et al. [19]	4 weeks p.o.	4 weeks p.o.

In the first group, roadworthiness is already reached two days following a minimally invasive THA [12], a week following a regular THA [7], two weeks following a regular THA [13], 3.7 weeks following a regular THA [8] and 6th + 7th weeks post-surgery in the present study. The results for TKA are similarly inconsistent: 4 weeks post-surgery [13], 4.4 weeks post-surgery [8], 6 weeks post-surgery [14], and in 6th +7th weeks post-surgery in the present study.

In a review study by Latz et al. a recommendation of 2 to 4 weeks of rest is made before resumption of driving after a TKA or THA [15]. Goodwin et al. on the other hand, recommend 10 days to 8 weeks of rest following a right-sided TKA and 6 to 8 weeks of rest following a right-sided THA before resumption of driving [16].

When it comes to the brake reaction time a similar picture is painted: Marques et al. recorded 44 ± 19 days for the patients to reach their preoperative brake reaction time following a right-sided TKA and 20 ± 15 days following a left-sided TKA [17]. Nizam et al., however, recorded a Median of 3 weeks following a TKA, regardless of side [18]. Van der Velden et al. and Huang et al. saw a return to the preoperative brake reaction time 4 weeks following a TKA and was able to show an inverse correlation between brake reaction time and the “step-test” by Marmon et al. [13, 19, 23].

There were several limitations related to our study. Since most patients with a TKA or THA undergo a three week bedridden rehabilitation in Germany, there is until the end of the 5th postop week, no reason to reuptake driving again. It could be considered that the bedridden rehabilitation impairs the patient to reuptake driving earlier than they actually do. This is a particularity of the German health care system and could be considered a limitation of this study, when compared with other national health care systems, where a bedridden rehabilitation after a TKA or THA is not a common practice. Also the fact that this is a single centre study and although the study sample size was calculated, with the help of a statistician, prior to study begin, it is too small to upscale this study results at the national level and only reflects the postoperative driving practices after TKA or THA of one region of Germany.

The large bandwidth of results across the board and the resulting variance of recommendations likely leave the patient at a loss. An operational approach which would examine the skills required for safe driving and base recommendations on these parameters would be more practical. A low pain level, sufficient range of motion (ROM) of the affected joint, unaided walking, safe entrance and exit from the vehicle, a preferably high score in the “step-test” and an acceptable brake reaction time seem to be the most crucial parameters to develop individual recommendations for patients. What role these parameters play in the decision-making process for the postoperative resumption of driving following a TKA or THA is unclear. Furthermore the patient self-evaluation after a TKA or THA procedure is, at least in Germany, the main criteria for making the decision when to resume driving again. Further research is required to examine the significance of these parameters in recommendations.

## Conclusions

The results of the present study show differences in the resumption of driving after surgery with respect to gender, place of residence, operated side and identification of risk sub-groups of late driving.

When making the decision of when to resume driving following a TKA or THA patients should be individually advised. During the three-week in-patient rehabilitation program mandated in Germany patients should be supported and educated. This may be specifically useful for female patients who have undergone TKA.

From the present data we can conclude that driving after THA or TKA may be possible and safe at 4 weeks. We recommend to integrate mandatory education programs for driving after TJA in the current rehabilitation phase.

## Supplementary Information

**Additional file 1.**

## Data Availability

The datasets during and/or analysed during the current study are available from the corresponding author on reasonable request.

## Abbreviations

TKA: total knee arthroplasty
THA: total hip arthroplasty
TJA: total joint arthroplasty
