# Supplementary material for: Surgical site, gender, and place of residence influence the time to resume driving after total joint arthroplasty
Source: Arch Physiother. 2021 Jun 29;11:16. doi: 10.1186/s40945-021-00111-4 (PMC8240401; doi:10.1186/s40945-021-00111-4)
Supplement: Supplementary file 1 — Additional file 1. [file 40945_2021_111_MOESM1_ESM.pdf]

Study Questionnaire

1. Patient number: ______________________
2. Do you posses a valid driver's license?
   - yes ☐
   - no ☐
3. Patient gender:
   - male ☐
   - female ☐
   - divers ☐
4. Patient age ______________________
5. What joint replacement procedure did you went through?
   - knee replacement procedure ☐
   - hip replacement procedure ☐
6. Which side was operated on?
   - right side ☐
   - left side ☐
7. Can you specify the date of the surgery?
   - Surgery date (DD/MM/YYYY) ______________________
8. Do you still use walking aids?
   - yes ☐
   - no ☐

9. How often did you drive a car prior to surgery?
   - daily ☐
   - 1-3/week ☐
   - occasionally ☐

10. Did you drive a car after your joint replacement surgery?
    - Yes – when?
      - 6th - 8th week after surgery ☐
      - 8th - 10th week after surgery ☐
      - 10th – 12th week after surgery ☐
    - No
      - I'm not feeling fit enough to drive ☐
      - my doctor recommended I shouldn't drive yet ☐
      - I am not driving the first 12 weeks after surgery ☐

11. Do you drive a manual or automatic transmission car?
    - automatic transmission ☐
    - manual transmission ☐

12. Do you still have joint pain after replacement surgery?
    - yes ☐
    - no ☐

13. Do you still have joint pain when you are pushing the car's pedals?
    - yes
      knee pain ☐
      hip pain ☐
      ohter location______________________________
    - no ☐

14. What was the reason for the first drive after surgery?
    - doctor/physical therapist appointment ☐
    - other reasons ☐
